# A Diatom Ferritin Optimized for Iron Oxidation but Not Iron Storage*

**DOI:** 10.1074/jbc.M115.669713

**Published:** 2015-09-22

**Authors:** Stephanie Pfaffen, Justin M. Bradley, Raz Abdulqadir, Marlo R. Firme, Geoffrey R. Moore, Nick E. Le Brun, Michael E. P. Murphy

**Affiliations:** From the ‡ Department of Microbiology and Immunology, University of British Columbia, Vancouver, British Columbia V6T 1Z3, Canada and; the § Centre for Molecular and Structural Biochemistry, School of Chemistry, University of East Anglia, Norwich Research Park, Norwich NR4 7TJ, United Kingdom

**Keywords:** algae, enzyme kinetics, ferritin, iron, protein structure, x-ray crystallography, ferroxidase center, iron storage mechanism

## Abstract

Ferritin from the marine pennate diatom *Pseudo-nitzschia multiseries* (PmFTN) plays a key role in sustaining growth in iron-limited ocean environments. The di-iron catalytic ferroxidase center of PmFTN (sites A and B) has a nearby third iron site (site C) in an arrangement typically observed in prokaryotic ferritins. Here we demonstrate that Glu-44, a site C ligand, and Glu-130, a residue that bridges iron bound at sites B and C, limit the rate of post-oxidation reorganization of iron coordination and the rate at which Fe^3+^ exits the ferroxidase center for storage within the mineral core. The latter, in particular, severely limits the overall rate of iron mineralization. Thus, the diatom ferritin is optimized for initial Fe^2+^ oxidation but not for mineralization, pointing to a role for this protein in buffering iron availability and facilitating iron-sparing rather than only long-term iron storage.

## Teaser

**Background:** Iron storage by ferritin enables diatom bloom upon iron input.

**Results:** Ferroxidase center variants show faster iron mineralization and rate of post-oxidation reorganization of iron.

**Conclusion:** Glu-130 and Glu-44 regulate the flux of iron through the ferroxidase center.

**Significance:** Optimization of ferritin for iron oxidation but not mineralization suggests an iron buffering function in addition to long-term iron storage.

## Introduction

Ferritins constitute a broad family of iron storage and detoxifying proteins found in all three domains of life (1). Typically formed from 24 subunits arranged as a symmetric, hollow sphere (2), ferritins take up soluble Fe^2+^ and catalyze its oxidation, with the resulting Fe^3+^ being stored as an oxy-hydroxide mineral in its central cavity (3).

Mammalian ferritins are composed of two different subunit types: H-chain and L-chain. The H-chain is associated with catalytic activity and contains a di-iron ferroxidase center, whereas the L-chain subunit contains putative nucleation sites for iron core formation (4).

Prokaryotes contain two subfamilies of 24-meric ferritins: the heme-containing bacterioferritins (BFRs)^5^ (5, 6) and the non-heme ferritins (Ftns) (1). Both comprise a single subunit type containing both the ferroxidase center and nucleation sites. Prokaryotic Ftns and eukaryotic ferritins form the archetypical subfamily of the ferritin family and have similar ferroxidase centers, whereas the ferroxidase center of BFRs is structurally distinct (1, 7).

Prokaryotic Ftns contain a third iron site, site C, located 6–7 Å away from the di-iron center. Site C is important in mineralization, although its precise role remains to be determined and may vary between ferritins from different species (8, 9).

Diatoms are unicellular photosynthetic eukaryotes that play an important role in primary production and carbon sequestration in the deep ocean (10, 11). A ferritin from the pennate diatom *Pseudo-nitzschia multiseries* (PmFTN) was shown to facilitate blooming after iron inputs in iron-limited regions of the ocean (12, 13). High resolution structures of PmFTN crystals soaked in aerobic ferrous iron solutions revealed iron bound at the ferroxidase center (sites A and B) and at a third site closer to the mineral core (13), demonstrating the first eukaryotic site C-containing ferritin. The PmFTN site C is located closer to the ferroxidase center than is the site C of prokaryotic Ftns but shares two common ligands (Glu-47 and Glu-130). Under anaerobic conditions, Fe^2+^ was observed bound only at site A (13). Initial Fe^2+^ oxidation was found to be rapid, first order with respect to the iron concentration, and saturated at 2 Fe^2+^ per subunit. A model was proposed in which Fe^2+^ ions bind stepwise in a dioxygen-dependent manner, with the binding of the second iron ion the trigger for oxidation to occur (12). However, the mechanism of sustained ferroxidase center turnover and transport of iron to the mineral core in PmFTN remains unknown and, in particular, the role of site C, as in other ferritins, is unclear. Kinetic studies involving sequential Fe^2+^ additions showed that iron does not completely vacate the ferroxidase centers following oxidation. Structural studies with crystals soaked for various durations revealed a partial mobilization of Fe^3+^ from the di-iron center to site C and sites further toward the central cavity, suggesting a complex iron transport mechanism that likely involves site C (13). In PmFTN crystal structures, Glu-130 is observed to coordinate iron at sites B and C, and Glu-44 is an iron ligand both at site C and on the inner surface of the protein shell. Glu-130 and Glu-44 are proposed to shuttle metal ions between these sites (13).

To define the function of site C in iron mineralization by PmFTN, variants of Glu-130 (E130A) and Glu-44 (E44H and E44Q) were characterized both functionally and structurally. The data reveal that Glu-130 is not required for rapid Fe^2+^ oxidation but functions to stabilize Fe^3+^ at the ferroxidase center, thereby greatly reducing the rate of mineralization. Glu-44 is shown to be important for regulating post-oxidation reorganization of iron coordination. Retention of iron at the ferroxidase center at the expense of mineralization points to a role for PmFTN in facilitating iron buffering rather than long-term iron storage. Finally, the observation of iron within the B-channels of the E44Q variant of PmFTN provides clear evidence that these channels, initially identified in prokaryotic ferritins and BFRs, are important routes for Fe^2+^ entry into the protein.

## Experimental Procedures

### 

#### 

##### Cloning of PmFTN Variants E130A, E44H, and E44Q

The site-directed PmFTN variants E44H and E130A were created by subcloning from the PmFTN wild type construct, a pET28a(+) vector containing the coding region of PmFTN genomic DNA lacking the signal peptide and plastid-targeting sequences (12). The expressed protein is lacking the proline at the N terminus and the valine at the C terminus as compared with the sequence found at UniProt entry B6DMH6.

The cloning method used for the E44H and E130A variant constructs was a modified whole plasmid polymerase chain reaction method (14). Briefly, to clone the E130A construct, a first PCR reaction synthesized megaprimers of ∼300 bp. The forward primer used for this reaction was 5′-CTTGTCTTCCGCGTTCACTTGTTG-3′, and the reverse primer was the T7-Terminator (5′-GCTAGTTATTGCTCAGCGG-3′). In a second PCR reaction, the whole plasmid was amplified using the megaprimers and the wild type PmFTN construct as a template. For the E44H variant, the megaprimer synthesis and whole plasmid amplification steps were combined into one PCR reaction, using the forward primer 5′-TCGCGTTCCTCCGCTGAATGTGCAAGCATGTAGGCGG-3′ and the T7-Terminator. The variant E44Q was synthesized by GenScript (Piscataway, NJ). All clones were verified by sequencing (Agencourt Bioscience, Beverly, MA).

##### Protein Expression and Purification

*Escherichia coli* BL21(DE3) cells were transformed with the appropriate expression vector (E44Q, E44H, or E130A PmFTN). The cells were inoculated into 2×YT medium supplemented with 25 μg/ml kanamycin and grown at 37 °C to an optical density of ∼0.8 at 600 nm. Protein expression was induced with the addition of 0.2 mm isopropyl β-d-thiogalactopyranoside. The protein was expressed at 25 °C overnight, and afterward, the cells were pelleted by centrifugation. The pellet was resuspended in 20 mm Tris-HCl, pH 8, 0.5 m NaCl, 1 mm TCEP, 5% glycerol (v/v), and 5 mm EDTA, and the cells were lysed at 4 °C using an EmulsiFlex-C5 homogenizer (Avestin, Ottawa, Ontario, Canada). Insoluble cell debris was removed by centrifugation. DNA was precipitated by the addition of 10 μl of 5% polyethyleneimine (w/v) per ml of supernatant. The reaction mixture was gently shaken for 10 min on ice, and afterward, the DNA was pelleted by centrifugation. PmFTN variants were purified using a heat shock method as described by Marchetti *et al.* (12). Briefly, the cell extract was aliquoted into 1-ml fractions, heat-shocked for 5 min at 60 °C, and put on ice for 4–5 min. The precipitated *E. coli* proteins were removed by centrifugation, and the supernatant was dialyzed against 20 mm Tris, pH 8, 5% glycerol (v/v), 5 mm EDTA and filtered through a 0.22-μm syringe filter. PmFTN variants were applied to a Source 15Q (GE Healthcare) column equilibrated in the same buffer and eluted using a 0–50% 1 m NaCl gradient. Purified PmFTN was dialyzed into 3% sodium dithionite (w/v), 1 m sodium acetate, pH 4.8, and 1 mm TCEP to remove bound iron to yield the apoprotein. Apo-PmFTN was further dialyzed into 50 mm MES, pH 6.5, 100 mm NaCl, and 1 mm TCEP (Buffer A). The cysteine residues were alkylated by first incubating PmFTN in Buffer A supplemented with 2 mm TCEP for 2 h at 37 °C with shaking. Iodoacetamide (10 mm) was then added, and the solution was incubated in the dark for 45 min at 37 °C with shaking.

##### Stopped-flow Absorption Spectroscopy

Rapid kinetic experiments were carried out using a stopped-flow instrument (Applied Photophysics DX17MV). Changes in absorption at 340 nm on the addition of Fe^2+^ to apo-PmFTN variants were measured. 10–400 μm Fe^2+^ working solutions were freshly prepared prior to each experimental run using a 50 mm stock solution of ferrous ammonium sulfate prepared in deoxygenated water, which was bubbled with argon gas for 2 h prior to use. The stock solution was acidified with 1 ml of 37% HCl per 100 ml of solution. For each variant, 1 μm apoprotein in 100 mm MES, pH 6.5, and 200 mm NaCl was mixed 1:1 by the stopped-flow instrument with the various Fe^2+^ working solutions, resulting in a protein concentration of 0.5 μm during data acquisition. All stopped-flow experiments were performed at 25 °C. As required, a single or double (or, in some cases, triple) exponential function was fit to the resulting data using the program Origin (version 8, OriginLab). Regeneration experiments were carried out using 1 μm PmFTN containing 48 iron ions, added under aerobic conditions during a fixed period (30 min or 20 h) prior to the stopped-flow experiment. As above, the appropriate function was fit to the resulting data. For additions of 400 Fe^2+^ per PmFTN, rates of Fe^2+^ oxidation were calculated from initial, linear increases in *A*_340 nm_ per unit time. Because ϵ_340 nm_ values could not be assumed to be constant between proteins, values of Δ*A*_340 nm_ min^−1^ values were converted to Fe^2+^ oxidized per min (μm min^−1^) (15).

##### Crystallization and Structure Solution

Crystals of PmFTN variants E44Q, E44H, and E130A were grown by hanging drop vapor diffusion at room temperature at a 1:1 ratio of protein to well solution (0.1 m sodium acetate, pH 5.5, 1–1.2 m ammonium sulfate, and 0.9–1.2 m sodium chloride). The protein was concentrated to ∼20 mg/ml in Buffer A supplemented with 2 mm TCEP and 10 mm iodoacetamide. The crystals were soaked in mother liquor supplemented with freshly prepared 2 mm ammonium ferrous sulfate hexahydrate for 5 min and overnight for the E130A variant, 5 min, 45 min, and overnight for the E44Q variant, and 45 min and overnight for the E44H variant. The crystals were transferred to a cryoprotectant consisting of mother liquor supplemented with 30% glycerol (v/v) before flash-freezing in liquid nitrogen.

PmFTN data sets were collected at the Stanford Synchrotron Radiation Lightsource on beamline 7.1 and at the Canadian Macromolecular Crystallography Facility of the Canadian Lightsource on beamline 081D-1 at 1 Å wavelength. Data were processed using Mosflm to resolutions of 1.8–2.0 Å. The data were merged using Scala in CCP4, and the resolution cut-off was determined by Mean((I)/S.D.(I)) and Mn(I) half-set correlation CC(1/2).

Phases were determined using MolRep (16) with a previously determined wild type PmFTN crystal structure (Protein Data Bank entry 4IWJ) as the search model after removal of the iron ions and solvent atoms (13). The initial model was edited in Coot (17) and refined with Refmac5 (18). Waters were added by running findwaters in Coot and refining in Refmac5. The variant protein crystals were isomorphous with crystals of wild type PmFTN, and the space group was either P42_1_2 or P23, with six or eight subunits in the asymmetric unit, respectively. Neither the amino acid substitutions nor the metal treatment altered the overall fold or the formation of the spherical structure. The refined structures had at most 3 or 12 residues absent from the N or C terminus, respectively. Ramachandran plot analysis showed that in all structures, more than 98% of the residues were in the preferred regions. Superposition of the variant subunits with wild type PmFTN resulted in a root mean square deviation of 0.28 Å or less for all Cα atoms.

Iron ions were added to the models as follows. Anomalous dispersion data at 1 Å wavelength were used to identify metal sites. Anomalous maps were computed using fft from the CCP4 package, using the model phases. Metal occupancies were fixed such that the B-factors were similar to those of the coordinating residues. If B-factors of some coordinating residues were outliers, metal occupancy was set such that the B-factor was similar to those of the residues with the shortest ligand bonds. Furthermore, occupancies were confirmed by inspection of peak heights in anomalous dispersion maps. Lastly, (*F_o_* − *F_c_*) difference maps were inspected to optimize the fit of the occupancy to the electron density. Peaks in anomalous maps were observed at the interface of the ferritin spheres of E130A and E44H variants, but electron density was not sufficiently well defined to model iron ions. Data collection and refinement statistics are shown in Table 1. Figures were generated with PyMOL (Version 1.7.2.2, Schrödinger, LLC).

**TABLE 1:** **Data collection and refinement statistics** ASU, asymmetric unit; o.n., overnight: ESU, estimated standard uncertainty; r.m.s.d., root mean square deviation.

	E44Q Fe (5 min)	E44Q Fe (45 min)	E44Q Fe (o.n.)	E44H Fe (45 min)	E44H Fe (3 h)	E130A Fe (5 min)	E130A Fe (o.n.)
**Data collection**							
Resolution range (Å)	48.56–1.80 (1.90–1.80)*^a^*	48.51–1.80 (1.90–1.80)	48.61–1.90 (2.00–1.90)	47.93–1.85 (1.95–1.85)	42.28–1.90 (2.00–1.90)	48.53–1.90 (2.00–1.90)	48.55–2.00 (2.11–2.00)
Space group	P23	P23	P23	P42_1_2	P42_1_2	P23	P23
Unit cell dimensions (Å)	*a* = *b* = *c* = 175.07	*a* = *b* = *c* = 174.92	*a* = *b* = *c* = 175.25	*a* = *b* = 126.46, *c* = 170.31	*a* = *b* = 126.72, *c* = 170.31	*a* = *b* = *c* = 174.98	*a* = *b* = *c* = 175.05
No. of subunits in ASU	8	8	8	6	6	8	8
Unique reflections	164,331	163,899	1403,66	117,178	108,379	139,649	120,022
Completeness (%)	100 (100)	100 (100)	100 (100)	99.6 (99.2)	99.3 (96.7)	100 (100)	100 (100)
Redundancy	14.3 (13.3)	14.0 (12.4)	13.4 (12.1)	6.7 (6.3)	8.6 (4.8)	14.3 (14.0)	13.9 (13.7)
Average I/σI	20.6 (2.9)	20.1 (2.9)	15.9 (2.5)	12.0 (2.1)	16.5 (2.5)	16.8 (2.5)	14.9 (2.4)
R-merge	0.085 (0.866)	0.082 (0.868)	0.110 (0.977)	0.083 (0.809)	0.077 (0.504)	0.110 (1.124)	0.107 (1.135)
Wilson B (Å^2^)	23.1	24.3	22.6	23.0	17.6	25.3	30.9
CC_Imean	0.826	0.791	0.779	0.646	0.756	0.710	0.670

**Refinement**							
R-work (R-free)	0.168 (0.201)	0.170 (0.205)	0.162 (0.199)	0.172 (0.207)	0.167 (0.204)	0.169 (0.208)	0.187 (0.227)
Avg. B (Å^2^)	27.2	28.4	28.1	29.5	28.4	31.2	41.1
No. of water molecules	985	902	875	699	707	881	510
No. of iron molecules	8	16	31	24	24	7	37
r.m.s.d. bond length	0.021	0.021	0.019	0.019	0.020	0.019	0.019
ESU from maximum likelihood (Å)	0.067	0.069	0.078	0.080	0.076	0.083	0.108
4ZKX	4ZKW	4ZKH	4ZL5	4ZL6	4ZMC	4ZLW	

*^a^* Values in parentheses for the data collection statistics are for the highest resolution shell indicated.

## Results

### 

#### 

##### Substitutions of Glu-44 and Glu-130 Disrupt Fe^2+^ Binding Cooperativity but Not Catalytic Activity

To explore the role of site C in iron mineralization by PmFTN, Glu-44 was substituted with glutamine and histidine, residues that occur naturally at this position in other characterized ferritins. Glutamine has a similar structure to glutamate but is not charged and does not usually coordinate iron ions. Histidine can also be neutral but is commonly found as a ligand for iron in metalloproteins.

**FIGURE 1.**
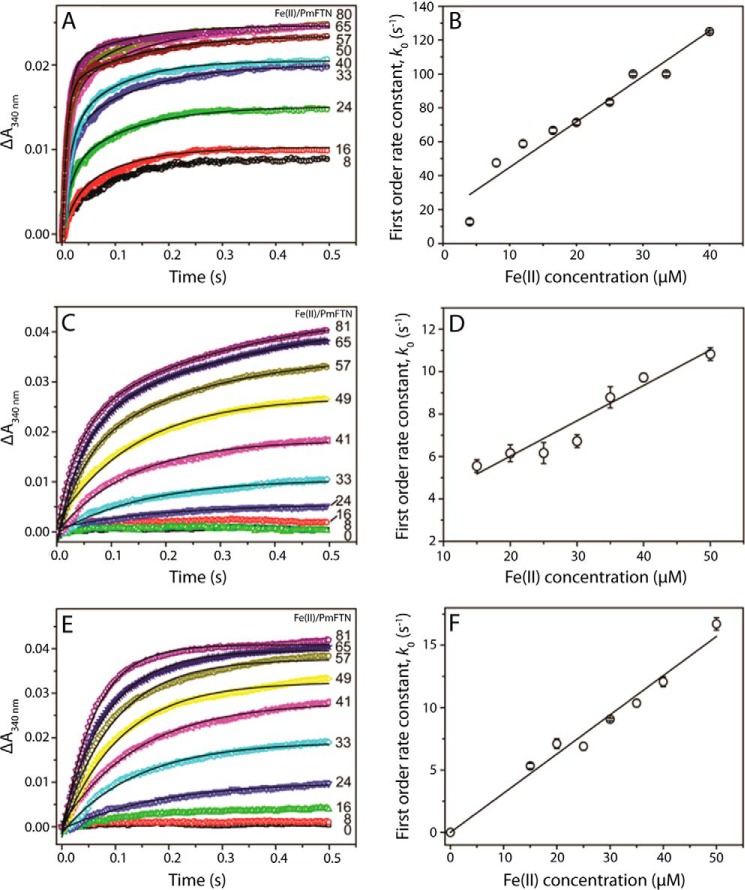
**Kinetic analysis of Fe^2+^oxidation catalyzed by E44Q PmFTN (*A* and *B*), E44H PmFTN (*C* and *D*), and E130A PmFTN (*E* and *F*).** A, *C*, and *E*, Fe^2+^ was added to final concentrations at increasing ratios as indicated to PmFTN variants (0. 5 μm final concentration) in MES buffer (0.1 m, pH 6.5, 25 °C). A double exponential function for *A* and *C* and a single exponential function for *E* were fit to each of the Δ*A*_340 nm_ traces (*solid line*). *B*, *D*, and *F*, plot of observed (pseudo-first order) rate constants for the initial oxidation reaction as a function of Fe^2+^ concentration. A linear fit of the data, giving the second order rate constant, is drawn in. *Error bars* represent the standard errors and for some data points lie within the circles.

Stopped-flow experiments were performed to monitor the kinetics of iron oxidation after Fe^2+^ additions to apo-PmFTN variants. Both Glu-44 variants were catalytically functional, and initial Fe^2+^ oxidation (measured as Δ*A*_340 nm_) occurred very rapidly (Fig. 1, *A* and *C*). In each case, a double exponential function was required to fit the data. A plot of observed (pseudo-first order) rate constants corresponding to the initial, rapid reaction as a function of Fe^2+^ concentration revealed a linear relationship demonstrating a first order dependence of the rate of oxidation on the concentration of Fe^2+^ (Fig. 1, *B* and *D*), as observed previously for the wild type protein (13). Apparent second order rate constants for the two Glu-44 variants, derived from the slope of lines of best fit, revealed significant differences. E44Q PmFTN has a rate constant ∼6-fold higher than that of wild type, whereas the rate constant for E44H PmFTN is about 3-fold lower than that of wild type PmFTN (Table 2).

**TABLE 2:** **First and second order rate constants of Fe^2+^ oxidation in PmFTN variants** N/A, not applicable.

	Wild type*^a^*	E44Q	E44H	E130A*^b^*
First phase (second order rate constant, m^−1^ s^−1^)	5.1 ± 0.1 × 10^5^	2.7 ± 0.3 × 10^6^	1.7 ± 0.2 × 10^5^	3.14 ± 0.01 × 10^5^
Second phase (first order rate constant, s^−1^)	0.13 ± 0.04	9 ± 1.4	0.50 ± 0.09	0.32 ± 0.07
Third phase (first order rate constant, s^−1^)	N/A	0.3 ± 0.1	N/A	N/A

*^a^* Note that there was an error in the calculation of the second order rate constant for the wild type protein (13); the plot of first order rate constant versus Fe^2+^ did not take into account the 1:1 mixing of the Fe^2+^ solution.

*^b^* The higher rate constant for the initial oxidation phase of E130A and the lower rate constant for the second phase of E130A compared with E44H PmFTN accounts for the clearer kinetic separation of the phases of E130A (Figs. 1 and 3).

Glu-130, a ligand that can coordinate iron at both site B and site C, was substituted with the non-coordinating residue alanine. A single exponential function fit well the Fe^2+^ oxidation kinetic data for E130A PmFTN. The same first order dependence on Fe^2+^ (Fig. 1*F*) was observed as for wild type protein and the Glu-44 variants, giving an apparent second order rate constant slightly lower than that of wild type PmFTN (Table 2).

**FIGURE 2.**
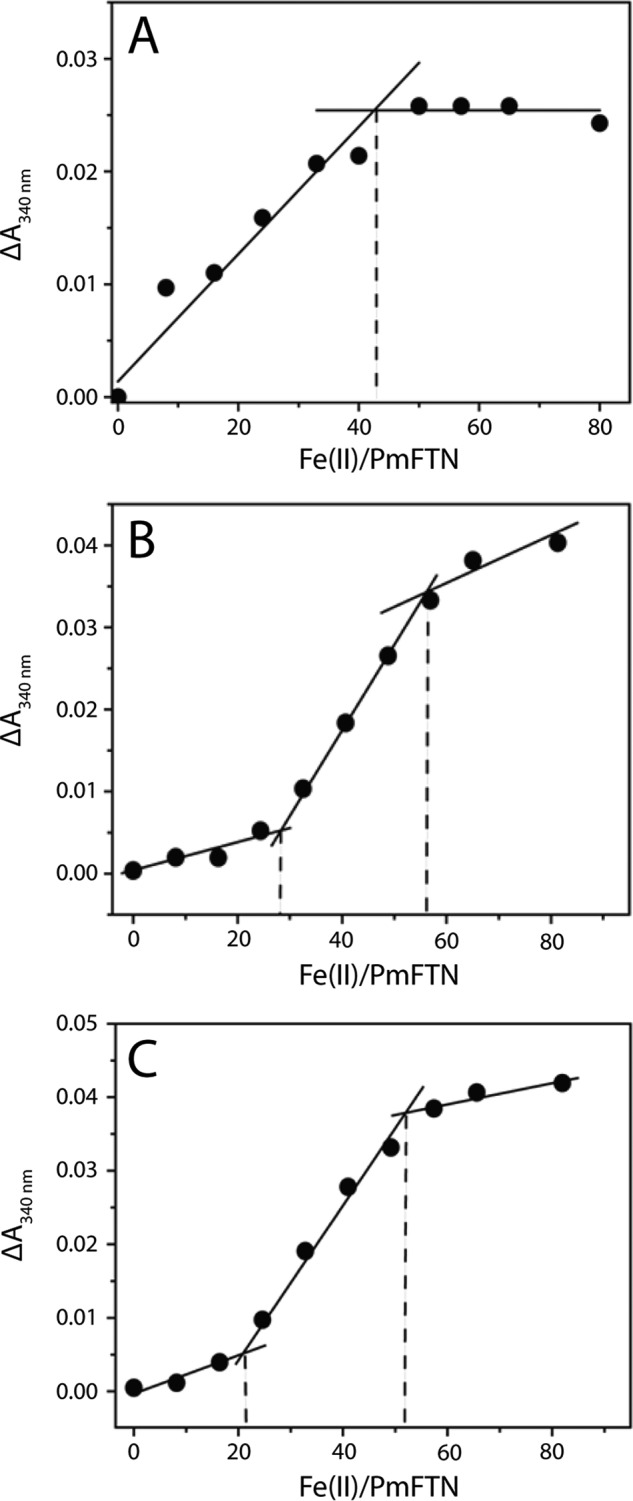
**Plots of total amplitude of absorbance changes at 340 nm at 0. 5 s as a function of Fe^2+^ added per PmFTN variant protein.** *A*, E44Q PmFTN. A change in absorbance can be observed after oxidation of up to ∼2 Fe^2+^ per subunit. *B*, E44H PmFTN. The incremental changes in absorption are small up to a level of ∼1 Fe^2+^ per subunit, increase up to ∼2 Fe^2+^ per subunit, and decrease again after ∼2 Fe^2+^ per subunit. *C*, E130A PmFTN. A similar absorption profile as for E44H PmFTN is observed.

A plot of Δ*A*_340 nm_ as a function of molar equivalent of Fe^2+^ added for the site C variant E44Q PmFTN (Fig. 2*A*) revealed saturation of the rapid oxidation phase at a level of ∼2 Fe^2+^ per subunit, indicating that the formation of oxidized di-iron sites is preferred over the formation of mono-iron sites. This cooperativity of Fe^2+^ binding/oxidation is similar to that of the wild type protein (13). Different behavior was observed for E130A and E44H PmFTN. Plots of Δ*A*_340 nm_ as a function of Fe^2+^ showed small absorption changes up to a level of ∼1 Fe^2+^ per subunit, after which they significantly increase, levelling off when all 24 subunits bound ∼2 equivalents of Fe^2+^, consistent with binding and oxidation of two Fe^2+^ per ferroxidase center (Fig. 2, *B* and *C*). The initial shallow slope is consistent with the requirement of double occupancy of the center by Fe^2+^ in order for oxidation to proceed. The low absorbance increase observed up to ∼1 Fe^2+^ per center indicates that Fe^2+^ binding does not occur pairwise as in the wild type protein, and we interpret this as the loss of positive cooperativity of Fe^2+^ binding and oxidation in these variants. This is replaced by either negative cooperativity, with binding of one Fe^2+^ ion at each ferroxidase center favored over double occupancy, or altered affinities of the two ferroxidase center sites such that one is preferentially occupied.

##### Enhanced Rate of Post-oxidation Reorganization in PmFTN Variants

**FIGURE 3.**
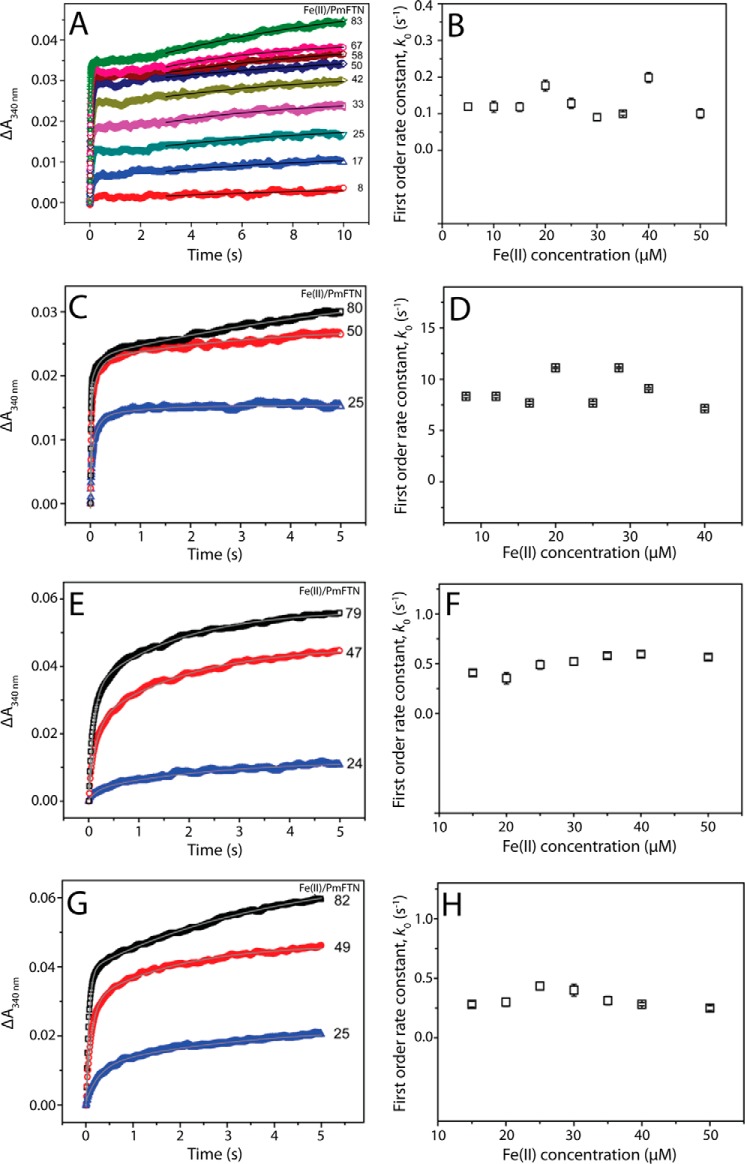
**Stopped-flow measurements of the second kinetic phase.** *A*, Fe^2+^ was added at increasing ratios to wild type PmFTN (0. 6 μm final concentration) in MES buffer (0.1 m, pH 6.5, 25 °C), and absorbance changes were followed over 10 s. A single exponential function was fit to absorbance changes following initial oxidation (from 3 s, *solid line*). *B*, plot of the first order rate constant corresponding to the second, slower phase as a function of Fe^2+^ concentration. *C*, absorbance measurements over a 5-s time period following the addition of Fe^2+^ to E44Q PmFTN (0.5 μm) at the ratios indicated. A tri-exponential function (*solid line*) was fit to the data. *D*, plot of the first order rate constant corresponding to the second, slower phase as a function of Fe^2+^ concentration. Data from *C* and equivalent experiments at other iron loadings were used. *E*, absorbance measurements over a 5-s time period following the addition of Fe^2+^ to E44H PmFTN (0.6 μm) at the ratios indicated. A double exponential function (*solid line*) was fit to the data. *F*, plot of the first order rate constant corresponding to the second, slower phase as a function of Fe^2+^ concentration. Data from *E* and equivalent experiments at other iron loadings were used. *G*, absorbance measurements over a 5-s time period following the addition of Fe^2+^ to E130A PmFTN (0.6 μm) at the ratios indicated. A double exponential function (*solid line*) was fit to the data. *H*, plot of the first order rate constant corresponding to the second, slower phase as a function of Fe^2+^ concentration. Data from *G* and equivalent experiments at other iron loadings were used.

For E44Q and E44H PmFTN, a second kinetic phase was observed following the initial rapid oxidation and was investigated further for all the variants as well as wild type PmFTN over a longer time period. Fig. 3, *A*, *C*, *E*, and *G*, show kinetic traces following the addition of increasing Fe^2+^ to wild type, E44Q, E44H, and E130A PmFTN, respectively. For E44H and E130A PmFTN, a double exponential function fit well to the data, and rate constants due to the second, slower phase were plotted as a function of Fe^2+^ (Fig. 3, *F* and *H*). For E44Q PmFTN, a double exponential function was used at lower iron loadings, but above 33 Fe^2+^/PmFTN, a tri-exponential function was needed to fit the data (Fig. 3, *C* and *D*). The third (slowest) phase (*k* = 0.3 ± 0.1 s^−1^) suggests that further reorganization occurs over a longer time period. For each variant, the second phase was independent of the Fe^2+^ concentration, indicating that this kinetic phase is a process that occurs subsequent to the oxidation of Fe^2+^ and the absorbance change is connected with some alteration in the coordination of Fe^3+^. The rate constant for this phase was substantially greater for the E44Q variant (Table 2).

Although a second kinetic phase was previously observed for wild type PmFTN, it was not characterized (13). Fig. 3*A* shows that rapid initial iron oxidation by wild type PmFTN was followed by a lag (more obvious at high iron loadings) before further absorbance changes occurred. Fitting an exponential function to the latter gave an Fe^2+^-independent rate constant lower than those of the variants and ∼75-fold lower than that of E44Q PmFTN (Fig. 3*B*, Table 2). Thus, perturbation of Fe^3+^ coordination following initial Fe^2+^ oxidation occurs more rapidly in these site C and site B/C variants, particularly in E44Q PmFTN.

##### E130A PmFTN Exhibits Significant Regeneration of the Initial Rapid Oxidation Phase

**FIGURE 4.**
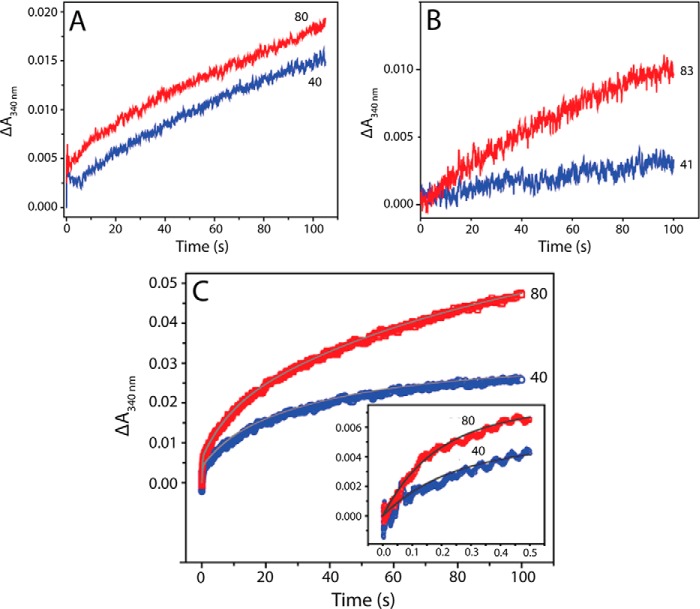
**Stopped-flow measurements of regeneration of the rapid oxidation phase.** *A*, measurement of absorbance changes at 340 nm following the addition of 40 or 80 Fe^2+^ per protein to a sample of E44Q PmFTN (0.5 μm after mixing) previously treated with 48 Fe^2+^ per protein under aerobic conditions. The incubation time between Fe^2+^ additions was 30 min. *B*, measurement of absorbance changes at 340 nm following the addition of 41 or 83 Fe^2+^ per protein to a sample of E44H PmFTN (0.6 μm after mixing) previously treated with 48 Fe^2+^ per protein under aerobic conditions. The incubation time between Fe^2+^ additions was 30 min. *C*, measurement of absorbance changes at 340 nm following the addition of 40 or 80 Fe^2+^ per protein to a sample of E130A PmFTN (0.5 μm after mixing) previously treated with 48 Fe^2+^ per protein under aerobic conditions. The incubation time between Fe^2+^ additions was 30 min. The *inset* shows the changes over the first 0.5 s. Fits to the data are shown as *solid lines*.

Flux of iron through the ferroxidase center following oxidation might lead to the regeneration of the rapid Fe^2+^ oxidation phase. Regeneration was not observed for the wild type protein presumably because the iron flux is too slow (13). PmFTN variants were loaded with 48 Fe^2+^ per 24-mer and subsequently incubated for 30 min. Then, a further aliquot of Fe^2+^ (40 or 80 Fe^2+^ per E130A or E44Q PmFTN; 41 or 83 Fe^2+^ per E44H PmFTN) was added, and changes in *A*_340 nm_ were measured (Fig. 4, *A–C*). Similarly to wild type protein (13), rapid oxidation was not observed in the Glu-44 variants. Remarkably, significant oxidation occurred in the E130A variant, and a tri-exponential function was required to fit the data. Importantly, the initial, rapid phase had a pseudo-first order rate constant (∼5 s^−1^) similar to that for the initial oxidation of Fe^2+^ measured in apo-E130A PmFTN (Fig. 1*F*). The amplitude of this phase indicated that ∼15% of ferroxidase centers of E130A PmFTN regenerated their apo form following the first round of Fe^2+^ oxidation. The second phase occurred with a rate constant (∼0.11 s^−1^) similar to the slower, iron-independent phase observed for the apoprotein. Similar data were obtained following an incubation period of 20 h (not shown).

##### E130A PmFTN Mineralizes Iron an Order of Magnitude Faster than Wild Type PmFTN

**FIGURE 5.**
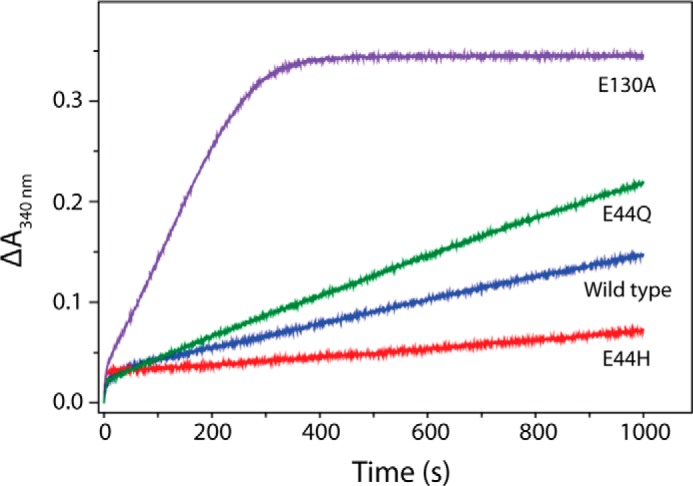
**Stopped-flow spectroscopy of iron mineralization in wild type and variant PmFTN.** Absorbance changes at 340 nm showing Fe^2+^ oxidation following the addition of 400 Fe^2+^/PmFTN to wild type and variant PmFTN (0.5 μm) in 0.1 MES, pH 6.5, at 25 °C are displayed.

Given the differences observed in the kinetics of post-Fe^2+^ oxidation and in the regeneration of initial rapid oxidation activity, the ability of the variants to mineralize iron was investigated. Iron core formation following the addition of 400 Fe^2+^ per apoprotein was followed by monitoring absorbance changes at 340 nm for 1000 s (Fig. 5), and initial rates of mineralization (*i.e.* post the rapid oxidation of two Fe^2+^ per ferroxidase center) were calculated. Mineralization in E44Q PmFTN was similar to wild type protein (initial rates of 7.0 ± 0.9 and 4.1 ± 0.8 μm Fe^2+^ min^−1^, respectively), whereas mineralization in E44H PmFTN was significantly slower (1.7 ± 0.5 μm Fe^2+^ min^−1^). Remarkably, mineralization in the E130A variant occurred more rapidly, with an initial rate of 40 ± 1 μm Fe^2+^ min^−1^, ∼10-fold faster than in wild type PmFTN.

##### No Iron Is Observed at Site C of E44Q PmFTN

**FIGURE 6.**
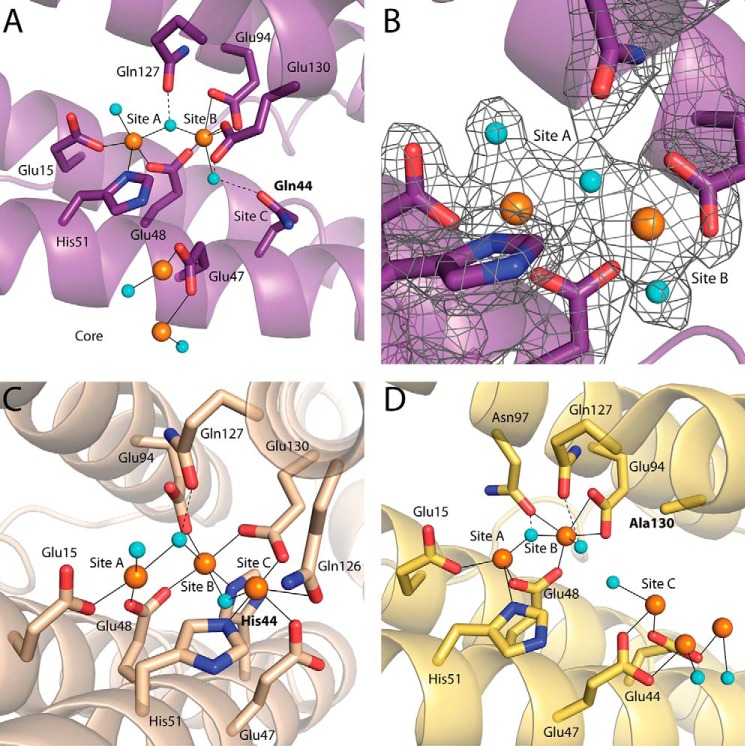
**Ferroxidase centers of PmFTN variants.** *A*, subunit B of the asymmetric unit of the E44Q Fe (o.n.) structure. Iron ions are bound to sites A and B, and two iron ions are bound at the inner surface. *B*, a *F_o_* − *F_c_* omit map contoured at 3 σ around site A and site B of the E44Q Fe (o.n.) structure is shown as a *gray mesh. C*, subunit A of the asymmetric unit of the E44H Fe (3 h) structure. Iron ions are bound to site A, site B, and site C. *D*, subunit D of the asymmetric unit of the E130A Fe (o.n.) structure. Iron ions are bound to sites A, B, and C. Two additional iron ions are bound past site C toward the mineral core. Iron ions are drawn as *orange spheres*, and *cyan spheres* represent water molecules. Side chains of selected residues are drawn in sticks with carbon, nitrogen, and oxygen atoms in the *backbone color*, *blue*, and *red*, respectively. *Solid lines* are metal ligand bonds, and *dashed lines* are selected hydrogen bonds.

Three crystal structures were obtained from E44Q PmFTN crystals soaked for 5 min (E44Q Fe (5 min)), 45 min (E44Q Fe (45 min)), and overnight (E44Q Fe (o.n.)) in an aerobic Fe^2+^ solution. The structure of the ferroxidase center of the latter is shown in Fig. 6*A*. Iron was observed bound at sites A and B with near full occupancy (Table 3), whereas site C was empty. As reported previously for wild type PmFTN (13), iron at site A (Fe-A) is coordinated by Glu-15, Glu-48, and His-51, and the iron ion at site B (Fe-B) is coordinated by Glu-48, Glu-94, and Glu-130. Fe-A and Fe-B are both coordinated by a terminal water molecule, with a third water bridging them. The bridging species could be an oxo/hydroxo group forming a diferric oxo/hydroxo bridge (13, 19). The water coordinated to Fe-B forms a hydrogen bond to Gln-44. Residue Glu-47 was part of site C in wild type PmFTN (13); however, in the E44Q variant structure, the side chain of this residue is instead pointing into the mineral core, and in five of eight subunits within the asymmetric unit, up to two iron ions are observed to be coordinated to Glu-47 (Fig. 6*A*). These iron ions were modeled at 25–35% occupancy and occupy distinct sites from those observed in wild type PmFTN. Reducing the Fe^2+^ exposure time of crystals to 5 and 45 min resulted in iron bound at sites A and B at lower occupancy, with the inclusion of Asn-97 in the coordination sphere for Fe-B. Site C remained unoccupied (Table 3, data not shown).

**TABLE 3:** **Range of iron ion occupancy observed in binding sites A, B, and C of aerobically Fe^2+^ soaked PmFTN variant crystals** —, not applicable.

	E44Q Fe (5 min)	E44Q Fe (45 min)	E44Q Fe (o.n.)	E44H Fe (45 min)	E44H Fe (3 h)	E130A Fe (5 min)	E130A Fe (o.n.)
Site A	20–25%*^a^*	45–55%	100%	55–65%	60–65%	0–35%	50–60%
Site B	—	20%	95–100%	95–100%	95–100%	—	0–35%
Site C	—	—	—	30–40%	40–45%	—	40–65%
Inner surface iron ions	—	—	0–35%	—	—	—	0–55%

*^a^* The metal occupancy was determined with an estimated error of ± 10%.

Two structures were obtained from E44H PmFTN crystals soaked for 45 min (E44H Fe (45 min)) and 3 h (E44H Fe (3 h)) in aerobic Fe^2+^ solution (Table 3 and Fig. 6*C*). All three sites are occupied by iron ions with modest differences in metal occupancy regardless of the crystal soaking time (Table 3). These structures revealed that the coordination spheres of the iron ions in sites A, B, and C are markedly altered in the E44H variant relative to the wild type PmFTN structure, exhibiting what might be described as ligand scrambling. The side chain conformation of His-51 is altered such that this residue now coordinates to the iron at site C (Fe-C) rather than Fe-A, which is thus coordinated only by Glu-15, Glu-48, a water molecule, and the bridging water or oxo/hydroxo group. His-44 and Glu-130 are ligands of Fe-B. Fe-C is coordinated by His-51 and Glu-130 and forms weaker interactions with Gln-126 and Glu-47. A water molecule is observed bridging Fe-C and Fe-B. As a result of these differences, Fe-C is displaced toward site A by ∼3.5 Å as compared with wild type PmFTN and the E44Q variant. However, the behavior of this variant cannot be readily interpreted in terms of a single residue substitution.

##### Iron Binding Site in the B-channels of E44Q PmFTN

**FIGURE 7.**
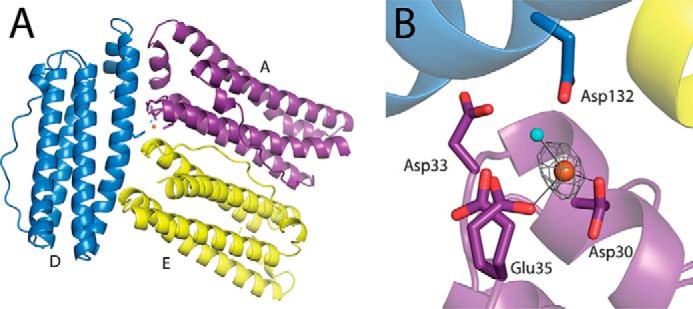
**Iron bound at a B-channel in the E44Q Fe (o.n.) structure.** *A*, a B-channel formed at the interface of subunits A (*purple*), D (*blue*), and E (*yellow*) as viewed from outside the ferritin sphere. *B*, the same B channel is presented in a close up view from the inner core surface. Side chains of selected residues are drawn in sticks with carbon and oxygen atoms in *backbone color* and *red*, respectively. *Solid lines* are metal bonds. An iron ion modeled at 25% occupancy and a water molecule are shown as *orange* and *cyan spheres*, respectively. An anomalous dispersion map contoured at 3 σ is shown as a *gray mesh*.

The presence of site C at the ferroxidase center of PmFTN suggests a higher degree of functional similarity to prokaryotic ferritins than previously observed for other eukaryotic ferritins. Similarity to prokaryotic ferritins extends to the presence of B-channels located at subunit interfaces where they connect the central cavity to the outer surface (20). These channels are proposed to function as routes for Fe^2+^ entry into the central cavity (20), but thus far, few experimental data exist to support this proposal. In E44Q Fe (o.n.), an additional iron ion was modeled within some of the B-channels (Fig. 7). The iron ion is close to the inner surface, coordinated by Glu-35, weakly coordinated by Asp-30, and coordinated by up to two solvent molecules, and modeled with occupancies of 25–35% (Fig. 7*B*). Glu-35 adopts two conformations, one coordinating to the iron ion and one pointing away from the channel. Iron was not found within the B-channels in structures from crystals with shorter soak durations.

##### Occupancy of Ferroxidase Center Fe-B Is Depleted in E130A PmFTN

Two structures were obtained from E130A PmFTN crystals soaked for 5 min (E130A Fe (5 min)) and overnight (E130A Fe (o.n.)) in aerobic Fe^2+^ solutions, respectively. In the E130A Fe (o.n.) structure, iron was found in binding sites A, B, and C (Fig. 6*D*). Iron occupancy at site A was 50–60%, whereas in site B, iron was observed in only six of eight subunits with an occupancy of 15–35%. Fe-C has an occupancy of 40–65% (Table 3). The iron coordination spheres of Fe-A and Fe-C are as in the wild type protein, except for the absence of the Glu-130 side chain. As seen in the E44Q Fe (45 min) structure (data not shown), Asn-97 is coordinated to Fe-B in the E130A Fe (o.n.) structure (Fig. 6*D*). Asn-97 is observed coordinating Fe-B or a water molecule modeled between the ferroxidase center sites A and B or at site B, depending on the subunit in the asymmetric unit.

In addition to these sites, up to two iron ions with occupancies of 40–55% were found beyond site C toward the mineral core, as seen in wild type PmFTN (13). One iron ion is coordinated by Glu-47, and the other is coordinated by Glu-44 and solvent molecules. The electron density for Glu-47 is weak, suggesting greater flexibility of this residue. The structure obtained from E130A Fe (5 min) has iron bound solely in site A (data not shown).

## Discussion

The presence of site C in bacterial and archaeal Ftns is a key feature that distinguishes them from mammalian H-chain ferritin and BFR (7). Site C was initially identified in *E. coli* FtnA (EcFtnA) and appears to function differently depending on the ferritin. In EcFtnA and *Pyrococcus furiosus* Ftn, the Fe^2+^:O_2_ ratio for the initial oxidation reaction is ∼3:1, suggesting that in these proteins, site C can participate in Fe^2+^ oxidation, with a mixture of H_2_O and H_2_O_2_ as the final product of O_2_ reduction (8, 9, 21, 22). In EcFtnA, site-directed mutagenesis of site C ligands Glu-49 or Glu-130 resulted in a decrease in oxidation rate and a drop in the Fe^2+^:O_2_ ratio to ∼2:1 (8, 9). Furthermore, these variants exhibited faster regeneration of the initial rapid oxidation phase, suggesting that the site may be important for controlling iron flux through the center (8). In *P. furiosus* Ftn, substitution of site C ligands led to complete loss of Fe^2+^ oxidation, indicating that the capacity of sites A and B to catalyze Fe^2+^ oxidation is dependent on site C (23). In PmFTN, the Fe^2+^:O_2_ ratio is ∼2:1, suggesting that site C does not function as a site of Fe^2+^ oxidation (12). Previous structural and kinetic data indicated that instead, site C functions as a transit site for iron from the ferroxidase center to the central cavity (13). The differing functional role of site C in PmFTN may be a consequence of its location and coordination sphere, which are distinct from those of bacterial and archaeal Ftns.

The rapid increase in absorbance at 340 nm upon the aerobic addition of Fe^2+^ demonstrates the binding of up two iron ions per ferroxidase center and their subsequent oxidation to Fe^3+^. In wild type PmFTN, this rate is exceptionally fast as compared with other characterized ferritins (13) (Table 2), and none of the three variants examined here were greatly diminished in this initial rate of ferroxidation. All exhibited the same first order dependence on Fe^2+^ concentration with saturation of this phase at ∼2 Fe^2+^ per ferroxidase center (13). Thus, rapid oxidation of Fe^2+^ at the ferroxidase center is not dependent on Glu-130 or on site C in general. Although overall kinetics of oxidation were not greatly affected, cooperativity of Fe^2+^ binding and oxidation was perturbed in E130A (and E44H) PmFTN such that significant Fe^2+^ oxidation only occurred above one Fe^2+^ per subunit. Structural data showed that, in both cases, iron at one of the sites (Fe-B in E130A PmFTN and Fe-A in E44H PmFTN) has fewer ligands than in the wild type structure, suggesting that inequivalent Fe^2+^ affinity at Fe-A and Fe-B accounts for the loss of positive cooperativity. However, at loadings >24 Fe^2+^ per PmFTN, this does not significantly affect the rate of ferroxidation.

Substitution of the site B/C coordinating residue Glu-130 with alanine, a non-coordinating residue, led to a striking 10-fold increase in mineralization activity; this is unusual in that active site mutations rarely enhance activity. Iron was observed bound to site B and site C in E130A PmFTN, showing that Glu-130 is not essential for iron binding at either site. However, crystals were soaked at relatively high iron concentrations, and these structures may not reflect iron occupancy under conditions used to assay mineralization rates.

In ferritins, such as human H-chain, in which the ferroxidase center functions as a gated site for the transfer of iron into the central cavity, the mineralization rate at lower iron loadings can be interpreted as a measure of the flux of Fe^3+^ through the center to the central cavity (24). Consistent with this model, partial regeneration of the initial rapid oxidation phase was observed in E130A PmFTN. The half-life for complete oxidation of the second Fe^2+^ addition was ∼5 s (as compared with >100 s for wild type PmFTN (13)). A longer incubation time did not affect the extent of regeneration, indicating that this is under thermodynamic control. Thus, Glu-130 plays a key role in regulating the flux of iron through the ferroxidase center, and we propose that it does this by coordinating Fe^3+^ at site B such that the rate of exit from the ferroxidase center is limited.

Structural data on wild type PmFTN showed that, as well as being a site C ligand, at longer soaking times, Glu-44 also coordinates iron ions on the inner surface and therefore appears to play a role in guiding iron toward nucleation sites for mineralization (13). Substitution of Glu-44 with glutamine resulted in the loss of iron binding at site C in iron soaking experiments. In this variant and after soaking overnight in Fe^2+^, Glu-47, which is a site C ligand in wild type PmFTN, was pointing away from the site, coordinating iron on the inner surface in a manner distinct from that observed in the wild type protein.

Previous kinetic studies revealed further changes in absorbance immediately following oxidation of Fe^2+^ at the ferroxidase center of wild type PmFTN, which were assigned to post-oxidation rearrangements of iron (13). For all of the variants, as well as the wild type protein, the rate of this subsequent phase was found to be independent of the initial Fe^2+^ concentration, consistent with the interpretation of a rearrangement of Fe^3+^ sites that caused changes in their electronic properties. In the E44Q variant, this rearrangement occurs ∼75-fold more rapidly than in the wild type protein (and ∼30-fold more rapidly than in the E130A variant). Thus, in the absence of a fully functional site C, post-oxidation rearrangement is significantly enhanced. The overall mineralization kinetics for E44Q PmFTN showed that this enhancement does not translate into a large increase in the rate of overall core formation. Presumably, this is because Glu-130 remains an Fe-B ligand, and therefore, a slower rate of iron transfer out of the center is retained. The nature of the post-oxidation rearrangement(s) is not clear. Absorbance at 340 nm is due to Fe^3+^-O charge transfer transitions; therefore, post-oxidation absorbance changes reflect changes in iron coordination. However, structural data suggest that there is no change of iron location, as Fe-A and Fe-B are fully occupied in E44Q PmFTN following overnight iron soak. We note that the solution and *in crystallo* experiments were carried out under different conditions and therefore are not directly comparable. If iron itself is not moving, then these changes are likely to be attributed to a reorganization of coordinating residues at the iron sites immediately following oxidation.

**FIGURE 8.**
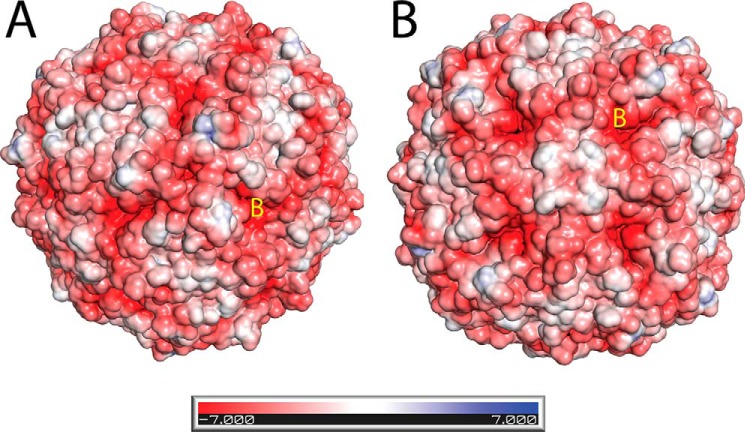
**Electrostatic surface potential of E44Q Fe (o.n.).** *A*, view down a three-fold channel. One of three visible B-channels is marked with a *yellow B. B*, view down a four-fold channel. One of four visible B-channels is marked with a *yellow B*. Negative and positive surface potentials are colored *red* and *blue*, respectively. The contouring value of the potential is in *kT*/*e*. Surface potentials were made using the PyMOL APBS tool.

Prior to structural studies of PmFTN, B-channels had only been observed in prokaryotic ferritin and BFRs. The conserved polar characteristics of these channels led to the suggestion that they are involved in transporting Fe^2+^ in or out of the central cavity (20). The E44Q Fe (o.n.) structure provides evidence that these channels can accommodate iron, coordinated toward the cavity end of the channel by Glu-35, Asp-30, and up to two water molecules. Fig. 8 shows the electrostatic surface potential of the E44Q Fe (o.n.) structure. Clearly, negative potential is visible at the entrance to the B-channels, consistent with a proposed role in Fe^2+^ transport. Interestingly, iron ions at the B-channels were not observed in the wild type protein or in any of the other variants, suggesting that binding of iron in the B-channels is enhanced in the E44Q variant. This conclusion is consistent with recent kinetic data on *E. coli* BFR, in which a B-channel variant was shown to have significantly reduced mineralization activity (25).

A remaining question is why PmFTN has evolved to oxidize Fe^2+^ at its ferroxidase centers so rapidly but to form an iron mineral so slowly. As a result, this protein is optimized to oxidize small amounts of iron extremely rapidly and to hold it at the ferroxidase center. This functional feature could indicate that, for some photosynthetic organisms, an iron buffering function may be more critical than long-term iron storage. For example, in the unicellular green alga *Chlamydomonas reinhardtii*, ferritin expression is not up-regulated under excess iron but rather under iron limitation, where it is involved in buffering iron as it is released from photosystem I by degradation (26–28). Such organisms may require the ability to rapidly re-model their iron proteome in response to limitation, and ferritin could play a key role in this function by holding iron at the ferroxidase center from where it may be more readily accessible to reductants for release as ferrous ions, as compared with when stored as an insoluble mineral in the protein cavity. PmFTN was shown to be important for the organism's ability to utilize transiently available iron in an otherwise iron-limited marine environment (12). Our data suggest that the protein's ability to maintain iron at the ferroxidase center, rather than to mineralize it, may be key to this role. Substantiating an iron buffering function will require developing methods to distinguish and measure rates of ferric iron reduction and release from the ferroxidase centers and the mineral core.

## Author Contributions

S. P., J. M. B., N. E. L. B., and M. E. P. M. designed experiments. S. P. performed the site-directed mutagenesis with the help of M. R. F. S. P. performed the protein expression, purification, and crystallization of all the PmFTN variants and solved all the crystal structures. S. P., R. A., and J. M. B. carried out the stopped-flow measurements. Regeneration and mineralization experiments were performed by R. A. and J. M. B. J. M. B., R. A., N. E. L. B., G. R. M., and S. P. performed stopped-flow data analyses. S. P., N. E. L. B., and M. E. P. M. wrote the paper with contributions from all co-authors.

## Footnotes

5 The abbreviations used are:

BFR: bacterioferritin
Ftn: prokaryotic non-heme ferritin
PmFTN: *Pseudo-nitzschia multiseries* ferritin
EcFtnA: *Escherichia coli* ferritin A
TCEP: tris(2-carboxyethyl)phosphine
o.n.: overnight.
